# The Infectivity of Enteric Fever

**Published:** 1904-02-27

**Authors:** 


### The Hospital.

A Journal of

The Medical Sciences and Hospital Administration.

Vol. XXXV.—No. 909. FEBRUARY 27, 1904.

### The Infectivity of Enteric Fever.

In dealing with the question of the channels of diffusion of enteric fever we have more than once had occasion to express the opinion that they were more numerous and more complicated than had been believed by many who had written upon the subject; and especially, in discussing the probable efficacy of Dr. Leigh Canney's proposals for the supply of boiled water to armies in the field, we pointed out the possibility, to say the least, that contaminated water was not the sole ordinary channel of conveyance, and that the bacillus might easily be deposited upon food by the agency of flies or of dust. The most convincing argument to the contrary was unquestionably furnished by Dr. Canney's own experience at Assouan, where boiled water was constantly given to the large body of labourers employed, and where the disease was entirely kept at bay. On the other side of the question we had the equally well-established fact that the urban water supplies of this country are now, as a rule, very effectively protected against pollution, with the result, on the one hand, that the prevalence of enteric fever has been greatly diminished, while, on the other, the disease has by no means disappeared. It has sunk to a lower level than that at which it stood when water pollution was a matter of frequent occurrence, but at that level it has remained, with comparatively slight variation from year to year. It has seemed to us certain that, in this country at least, some other agency than that of polluted water must be in common operation, and that the existing improved sources of supply had indeed simplified the problem, but had failed to solve it. A very important contribution to the question has just been made by Dr. Collie, whose unequalled experience of enteric fever invests his observations with a very high degree of authority, and who has published a pamphlet on what he describes as the personal infectivity of the disease. He maintains that it is frequently, or even commonly, diffused by direct infection, like small-pox or scarlet fever, and he cites a large number of instances in which it has attacked the nurses in charge of the enteric wards of the hospitals of the Asylums Board, who, he maintains, have contracted the disease by direct infection from the patients upon whom they were in attendance, and with whom they were brought into close personal relations, changing their linen, making their beds, washing their bodies, giving their food, removing their dejecta, and so on.

Dr. Collie declares that he has no intention of maintaining that enteric fever may not be conveyed by means of water, but only that in some instances this has not been established, and that, having regard to the evidence in favour of infection by personal intercourse recently brought forward, he believes that a case for the revision of the etiology of the disease has been made out. It would indeed be impossible to ignore the classical researches of William Budd into the diffusion of the disease along water channels in rural districts, or the analogies furnished by the progress of several epidemics of cholera; but Dr. Collie submits the evidence in favour of polluted water in certain outbreaks to very rigid examination, and returns at least a verdict of "not proven" in some that had been regarded as typical illustrations of this method of conveyance. He especially selects the report made by the late Sir George Buchanan upon an outbreak which occurred in Caius College in 1873; and shows not only that the suggestion of a fouled water supply in the College rests very much upon conjecture, and upon the assumption that something of the kind must have existed and had only to be sought for until it was found, but he also shows that the disease had been prevalent in the town of Cambridge for a considerable period, and that the possibilities of direct infection were numerous. The records of the earlier epidemics at Festiniog and at Wicken-Bonant are subjected to similar examination; and it will be conceded, we think, even by the most convinced believers in the universality and potency of water pollution, that the evidence in favour of its having existed in the places mentioned has been greatly shaken. Interesting details are also given with regard to the drain construction of the hospitals of the Asylums Board, so as to show the impossibility of any leakage of sewer air into the wards as a source of danger to the nurses. The great diminution in the prevalence of enteric fever in this country which has been witnessed during recent years was at first held to justify a hope of its eventual disappearance; and this hope, which has been much weakened by the arrest of the diminution at a certain point, may reasonably be revived if new channels of diffusion can be shown to be in existence. If Murchison can be proved to have been in error in his conclusion that the fever was not communicable from person to person, and if a further control over its diffusion can be established by limitation of personal intercourse with the sick, the prospects of its ultimate disappearance will be revived. As we said last week, much light is likely to be thrown upon this and other related problems by the military experience of Japan; and, in the meanwhile, Dr. Collie is entitled to the gratitude of the community for the force and clearness with which he has drawn attention to certain facts, and to the interpretations of which he thinks them to be susceptible.

